# Use of Adjunct Oral Methotrexate in the Management of Keloid Formation Following Syndactyly Revision Surgery: A Case Report and Review of the Literature

**DOI:** 10.7759/cureus.96978

**Published:** 2025-11-16

**Authors:** Alice Gargan, Cathryn Sprenger, Alya Abdul-Wahab, Andrew Fleming

**Affiliations:** 1 Department of Plastic and Reconstructive Surgery, St George's University Hospitals NHS Foundation Trust, London, GBR; 2 Department of Dermatology, St George's University Hospitals NHS Foundation Trust, London, GBR

**Keywords:** congenital hand surgery, incomplete syndactyly, keloid scar, oral methotrexate, paediatric plastic surgery

## Abstract

Keloid formation following elective syndactyly surgery is an uncommon but difficult complication to manage. This report presents a case in which oral methotrexate was successfully used as an adjunctive postoperative therapy to prevent keloid recurrence in a paediatric patient. A 12-month-old Somali girl developed extensive, treatment-resistant keloids after syndactyly release of the second web space on her right hand. Conventional treatments with silicone gel and intralesional triamcinolone proved ineffective. Surgical excision of the keloids and reconstruction with full-thickness skin grafts from the groin were followed by nine months of adjunctive oral methotrexate (0.4 mg/kg weekly), which was well tolerated and resulted in successful healing without recurrence. Methotrexate, a well-established antimetabolite used in autoimmune disorders, appears to influence the wound healing process by reducing fibroblast proliferation, modulating collagen synthesis, and controlling inflammation through adenosine pathway activation. In this case, the combination of surgery, grafting, and systemic methotrexate therapy effectively prevented keloid recurrence while maintaining hand function. Compared with traditional topical or intralesional approaches, methotrexate offers a systemic and relatively safe method of modulating the pathological mechanisms underlying keloid formation. However, existing evidence remains largely anecdotal, underscoring the need for larger, controlled studies to determine standardised dosing, treatment duration, and long-term safety in keloid prevention.

## Introduction

Keloid formation is characterised by an abnormal healing process, resulting in excessive scarring beyond the boundaries of the initial wound [1]. The exact cause of keloid formation is unclear. The proposed mechanism is thought to occur due to an imbalance between an increased synthesis of collagen and extracellular matrix, and decreased degradation of these products. Keloid lesions are characterised by excessive collagen, in the form of discrete nodules, and an excess of microvessels, most of which are partially or totally occluded due to an excess of endothelial cells [2]. The incidence of keloid scarring is higher among dark-skinned populations [3]. There are known anatomical sites that have a higher risk of keloid formation, such as the pre-sternal area, the back, and the posterior neck [4-7]. This is most likely due to mechanical stress, which is a known pathophysiological mechanism of the formation of keloid [2]. Moderately susceptible areas include the ears, upper arm, and anterior chest. It is important to note that keloids are rare on sites of lax skin, such as the upper eyelids, penis, scrotum, and hands and feet [4].

Syndactyly is one of the most common congenital anomalies of the hand, characterised by partial or complete fusion of adjacent digits. It may present as a simple form involving only soft tissue, or a complex form when osseous or nail structures are also fused. The condition can occur sporadically or as part of syndromic associations such as Apert or Poland syndrome. Clinically, syndactyly is evident at birth and has functional and cosmetic considerations. Diagnosis is primarily clinical, supported by radiographic imaging to assess the degree of skeletal involvement and guide surgical planning. The standard of care is operative management, typically performed in early childhood to optimise hand growth and function while minimising web creep and contracture.

Keloid formation following syndactyly release surgery is a rare complication with only a few previously reported cases [3-5,8-16]. This surgical complication can have a devastating impact on the range of movement and function in the digits of children. Sang et al. aimed to identify the predictive factors of keloid formation, in order to try to identify at-risk cases and initiate measures to possibly prevent the formation of keloids prior to undergoing syndactyly release surgery [11]. They performed a retrospective case-control study and found that patients with distal phalangeal bony enlargement and protrusion are at risk for keloid formation after syndactyly division. If the distal phalanx shows digital enlargement when compared with the adjacent digit, the risk of keloid development should be communicated preoperatively to the patients, and preventive strategies for keloid development and close observation are suggested. Keloid formation after syndactyly surgery is a rare but difficult complication. This case aims to highlight the successful use of oral methotrexate as an adjunctive therapy for preventing keloid recurrence in a paediatric patient.

## Case presentation

**Figure 1:**
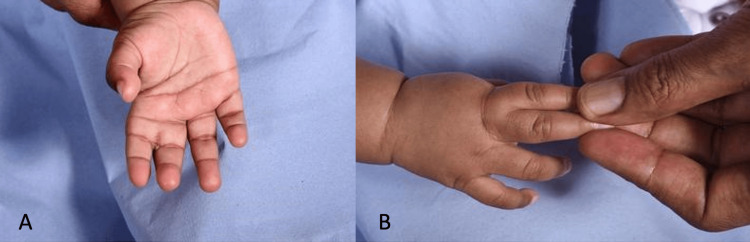
Incomplete syndactyly of the second web (A: volar view, B: dorsal view)

**Figure 2:**
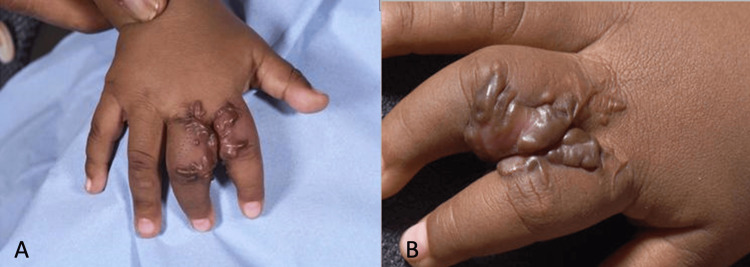
Keloid scar formation 18 months following initial surgery (A: dorsal view, B: closer dorsal view)

We report the case of a 12-month-old Somali girl who presented with incomplete syndactyly involving the second web space of the right hand (Figure 1). She was otherwise healthy, with no family history of keloid formation or congenital limb anomalies. The patient underwent elective syndactyly release to improve hand function and allow normal digital development. Postoperatively, the initial wound healing appeared satisfactory; however, within several months, she developed large, firm, raised scars extending beyond the surgical margins (Figure 2). The lesions were pruritic, hyperpigmented, and progressively enlarging, consistent with keloid formation. These keloids resulted in mild limitation of finger abduction and parental concern regarding both functional restriction and aesthetic appearance.

**Figure 3:**
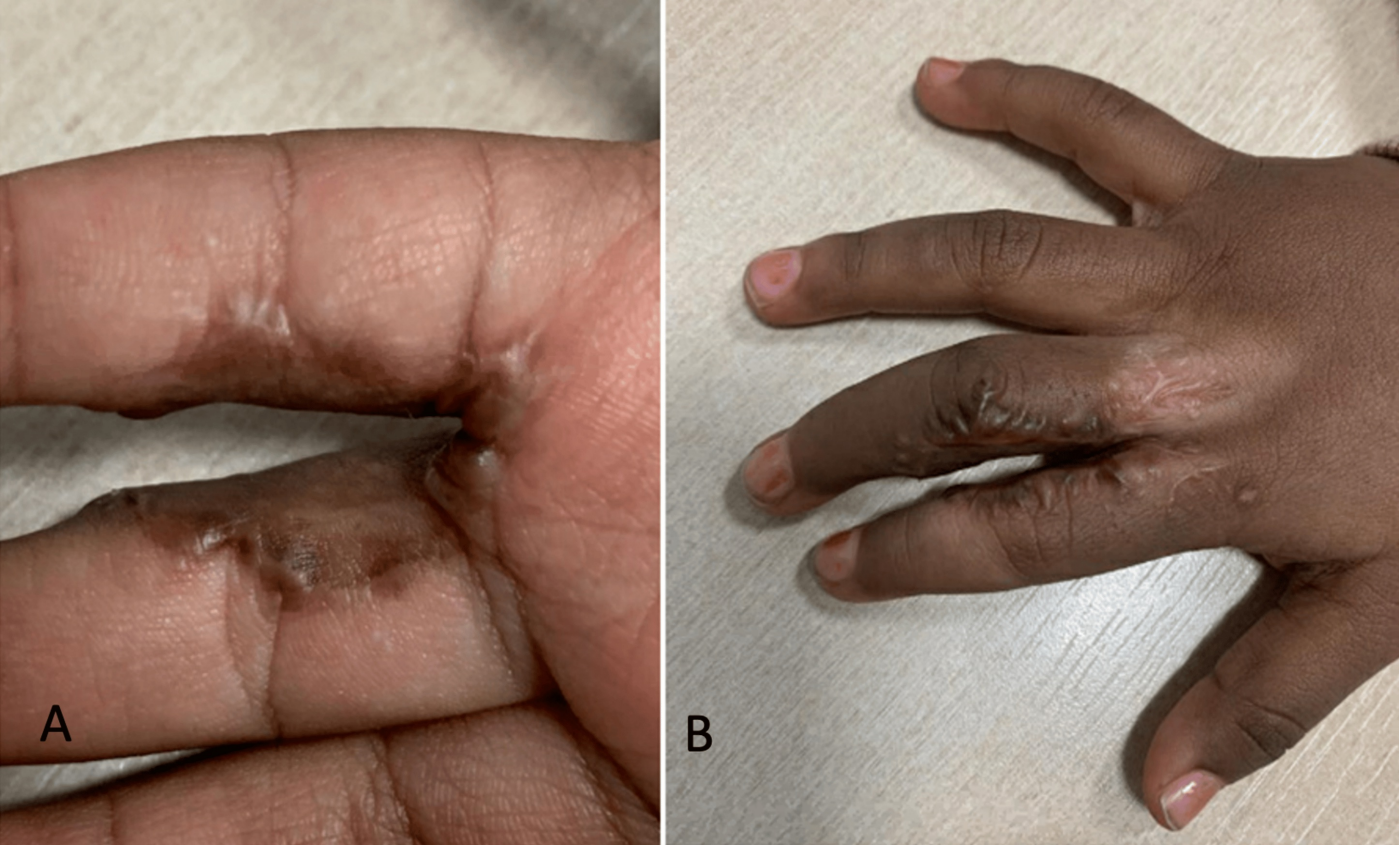
Nine months post keloid excision, full-thickness skin grafts and adjunct oral methotrexate (A: volar view, B: dorsal view)

Initial management with silicone gel sheeting and intralesional triamcinolone injections was unsuccessful. Given the refractory nature of the keloids, the lesions were surgically excised, and the resultant soft tissue defects were reconstructed with full-thickness skin grafts harvested from the groin. To minimise the risk of recurrence, adjunct oral methotrexate therapy was initiated postoperatively at a dose of 0.4 mg/kg weekly and continued for nine months. At nine months follow-up, the surgical site exhibited excellent healing, with supple, pliable skin, no keloid recurrence, and restoration of hand function (Figure 3). The donor site healed uneventfully, and methotrexate was well tolerated by the patient.

## Discussion

The use of oral methotrexate as an adjunct therapy to prevent keloid formation, particularly after syndactyly release surgery, is an emerging area of interest in reconstructive and paediatric hand surgery [7,16]. Keloid formation poses significant challenges in postoperative management due to its unpredictable nature, high recurrence rates, and resistance to conventional treatments. Traditional therapies, including steroid injections, silicone sheeting, compression, and radiation therapy, have been commonly employed to reduce keloid formation. There is limited evidence on the efficacy of different methods, which provide inconsistent results and may not be feasible in paediatric patients due to side effects and logistical challenges. As a result, methotrexate, an antimetabolite and immunomodulatory drug traditionally used in autoimmune conditions, is gaining attention as a preventive measure against keloid formation in high-risk cases.

Methotrexate exerts its effects primarily by inhibiting dihydrofolate reductase, thereby disrupting DNA synthesis and cellular replication, which is especially effective in highly proliferative tissues such as keloid-prone scar tissue [7]. At lower doses, methotrexate has also been shown to activate adenosine pathways, which helps to reduce inflammation by diminishing leukocyte recruitment, inhibiting stimulated neutrophil adhesion to vascular endothelium, and neutrophil-mediated injury to the endothelium [17]. Keloid formation is driven by an abnormal, exaggerated wound-healing response that involves the proliferation of fibroblasts, excessive collagen deposition, and persistent inflammation. Methotrexate's dual action of dampening cell proliferation and reducing inflammation could help to normalise wound healing and prevent excessive scar formation.

Few case reports and small observational studies have documented methotrexate’s role in keloid prevention following syndactyly release. Muzaffar et al. noted that paediatric patients with underlying conditions like macrodactyly and primary digital enlargement, both considered risk factors for keloid formation, were successfully managed surgically, followed by adjunctive prophylactic low-dose methotrexate therapy [5]. In this small cohort, no keloid recurrence was observed over a follow-up period of one year, suggesting a potentially protective effect of methotrexate in cases with high keloid risk. This study provides an important foundation for understanding the potential utility of methotrexate in managing keloid-prone paediatric cases.

An observational study by Tolerton and Tonkin described a cohort of children with complex syndactyly and macrodactyly who underwent syndactyly release surgery and were subsequently treated with oral methotrexate [3]. Their findings were promising, indicating that methotrexate might help in preventing or minimising the formation of keloids in paediatric patients predisposed to abnormal scar formation. However, this study also highlighted the lack of standardisation in methotrexate dosing and administration timing, which limits the reproducibility of the results across different patient populations and surgical settings.

While preliminary studies show promise, the evidence supporting methotrexate’s effectiveness in preventing keloids is not universally positive. Kong et al. reported on a series of cases where methotrexate was used postoperatively in paediatric patients with syndactyly [1]. In this study, methotrexate successfully prevented keloid formation in some patients, while others still developed keloids, although the keloids tended to be less aggressive in those treated with methotrexate. These mixed results suggest that methotrexate’s efficacy may depend on individual patient characteristics, including genetic factors, immune response, and possibly the extent of pre-existing tissue anomalies like macrodactyly. The authors noted that additional research is needed to determine the optimal dosing and timing of methotrexate therapy to maximise its efficacy in keloid prevention [1].

Comparative studies examining methotrexate against other keloid-preventive therapies are limited, but some reports offer indirect insights. Rosen et al. implemented a protocol combining surgical excision with intraoperative and postoperative steroid injections for ear keloids, achieving an 80% success rate over a minimum of five years of follow-up [14]. Shin et al. conducted a meta-analysis comparing triamcinolone injections and radiation therapy post-ear keloid excision. Both treatments had similar recurrence rates (15.4% for triamcinolone and 14.0% for radiation), indicating comparable efficacy [15]. However, the applicability of these treatments in paediatric patients is limited due to potential side effects and the need for repeated interventions. Unlike intralesional steroids, oral methotrexate offers a systemic approach that helps reduce the need for frequent interventions and provides a more tolerable alternative for children and their families.

Laspro et al. discuss the use of radiation therapy as an adjunct for preventing keloids, which has shown efficacy but is controversial, especially in paediatric populations due to the potential long-term risk of radiation exposure [18]. Methotrexate, by contrast, does not carry such risks, and its well-documented safety profile in children with autoimmune disorders supports its application in surgical cases, especially with the appropriate dosing and monitoring.

Methotrexate has a favourable safety profile when used in low doses, which has been established in paediatric populations for various autoimmune and inflammatory conditions [19]. Current guidelines recommend methotrexate doses up to 1 mg/kg per week in paediatric patients, with a maximum of 25 mg weekly. Common side effects, such as gastrointestinal discomfort and mild hepatotoxicity, are generally manageable and reversible. The absence of severe adverse events in studies involving low-dose methotrexate for keloid prevention suggests that this therapy is well-tolerated, although close monitoring is necessary to mitigate any potential risks. However, the lack of standardised protocols for methotrexate dosing, timing, and duration in keloid prevention is a significant limitation in its application. In the studies reviewed, dosing ranged widely from a single intraoperative dose to several weeks of postoperative administration, highlighting the need for more precise dosing recommendations tailored to keloid prevention rather than the management of inflammatory conditions.

## Conclusions

The current literature on methotrexate for keloid prevention is limited to case reports and small observational studies, with a lack of randomised controlled trials to validate findings. Many studies are complicated by additional treatments such as steroid injections and silicone sheeting, which makes it challenging to isolate methotrexate's effects. In addition, most reports have short follow-up periods, limiting the ability to assess long-term keloid recurrence. Furthermore, the heterogeneity in dosing regimens and patient selection criteria across studies prevents the development of standardised protocols, and the small sample sizes reduce the statistical power of existing findings. Although methotrexate is not yet widely accepted or standardised as a preventive treatment for keloid formation following syndactyly release, the available literature suggests potential benefits, particularly in patients with high-risk characteristics. Methotrexate offers a relatively safe and systemic approach to modulating the abnormal wound healing processes that contribute to keloid formation, distinguishing it from conventional topical and intralesional treatments. Future research should focus on conducting well-designed randomised controlled trials to establish optimal methotrexate dosing, timing, and duration specific to keloid prevention in surgical settings, in both adults and the paediatric population. In addition, research on patient-specific factors, such as genetic predispositions and immune response markers, could help tailor methotrexate therapy to maximise efficacy and minimise recurrence. As the body of evidence grows, methotrexate may emerge as a viable and effective adjunct therapy for preventing keloid formation in paediatric patients undergoing syndactyly release, particularly those at high risk of abnormal scar formation.
